# First Isolation and Molecular Characterization of Chicken Astrovirus and Avian Nephritis Virus in Chickens in Bangladesh

**DOI:** 10.3389/fvets.2021.769489

**Published:** 2021-12-01

**Authors:** Md Zulfekar Ali, Mohammad Moktader Moula, Zafar Ahmed Bhuiyan, Md Giasuddin, Hyun-Jin Shin

**Affiliations:** ^1^Animal Health Research Division, Bangladesh Livestock Research Institute, Dhaka, Bangladesh; ^2^Nourish Central Poultry Laboratory, Nourish Poultry and Hatchery Ltd., Dhaka, Bangladesh; ^3^College of Veterinary Medicine, Chungnam National University, Daejeon, South Korea; ^4^Research Institute of Veterinary Medicine, Chungnam National University, Daejeon, South Korea

**Keywords:** avian nephritis virus, CAstV, chicken astrovirus, ANV, poultry, Bangladesh

## Abstract

Chicken astrovirus (CAstV) and avian nephritis virus (ANV) are enteric viruses of poultry and have infected a wide range of poultry species worldwide, causing runting-stunting syndrome (RSS), which requires virus screening and results in serious economic damage. No confirmed cases have been reported from Bangladesh. In the present study, CAstV and ANV were monitored in Bangladesh. We monitored samples for CAstV and ANV and compared their genomic sequences to other reference strains. We found 8/31 flocks (25.8%) were positive for CAstV, 6/31 flocks (19.3%) had mixed infection of CAstV and ANV, and 1 flock (3.2%) was positive for ANV. Only ANV and a combination of CAstV and ANV were found in broilers and broiler breeders, but CAstV was found in all types of chickens. We isolated two of each from CAstV and ANV through specific pathogen-free chicken embryonated eggs via the yolk sac route. Phylogenetic analysis based on the ORF1b conserved region of CAstV and ANV suggested that the locally circulating strain was closely related to the strains isolated from India and Brazil. This report is the first molecular characterization of CAstV and ANV in Bangladesh. This study highlights that CAstV and ANV are circulating in Bangladeshi poultry.

## Introduction

Good enteric health plays a critical and basic role in successful broiler production by converting feed into meat. Any disturbances in this conversion process, such as mechanical, chemical, or biological hazards, impair nutrient absorption rates, resulting in high economic losses because of poor growth, increased mortality rates, and increased treatment costs (1). The major enteric viruses, including chicken astrovirus (CAstV), avian nephritis virus (ANV), avian rotavirus (AvRV), chicken parvovirus (ChPV), infectious bronchitis virus (IBV), avian reovirus (ARV), and fowl adenovirus (FAdV), are etiologies of enteritis of poultry that are accompanied by high economic losses to the poultry industries (2–5). Enteritis results in the alteration of the gastrointestinal environment and a direct decrease in feed digestion and absorption, consequently elevating the feed conversion ratio (FCR), causing growth retardation [commonly known as runting-stunting syndrome (RSS)], and immunosuppression, and an increased flock mortality rate by influencing secondary infections of the gut commensals (6–9).

RSS is relevant to the gastrointestinal health of poultry species and plays a significant role in the chicken meat industry and poultry health management because multiple viruses are involved (4, 10, 11). RSS is also called malabsorption syndrome, infectious stunting syndrome, helicopter symptoms, uneven growth, and malassimilation (11–17). Avian astrovirus (AAstV) belongs to the family *Astroviridae* and genus *Avastrovirus*. To date, six different astroviruses have been identified in avian species based on the species of origin and viral genome characteristics: two turkey-origin astroviruses [Turkey Astrovirus type 1 (TAstV-1) and type 2 (TAstV-2)]; two chicken-origin astroviruses [Avian Nephritis Virus (ANV) and Chicken Astrovirus (CAstV)]; and two duck-origin astrovirus [Duck Astrovirus type 1 (DAstV-1) and type 2 (DAstV-2)]. ANV has also been detected in turkeys, ducklings, pigeons, and guinea fowl; and TAstrovirus-2-likeviruses have also been found in guinea fowl (18). The genotypic classification, nine avastrovirus genotypes have been reported so far (AAstV-1-9). AAstV-2, -5, -8, -9 (typical chicken ANV) and AAstV-4 (chicken astrovirus, CAstV), are more frequently detected in chickens. There are some uncommonly described astroviruses in chickens such as AAstV-5 (or pigeon ANV-1 detected only in Chinese chickens) and AAstV-1 (TAstV-1 detected only in chickens from Sri Lanka) (19–21).

Morphologically, an astrovirus has a 7-kb-long single-stranded positive-sense RNA and it is nonenveloped, rounded, and 28 to 30 nm in diameter. Before their molecular characterization, astroviruses were thought to be Picornavirus or enteroviruses because of a typical star-like round morphology and size (22). The avian astrovirus genome contains three overlapping open reading frames, including ORF1a, ORF1b, and ORF2. ORF1a and ORF1b encode the viral protease and polymerase, respectively. ORF2 encodes a VP90 capsid precursor protein that is expressed from a subgenomic RNA (17).

An astrovirus was first detected in a young patient suffering from diarrhea and it has been reported as a causative agent of gastrointestinal disorders in humans and animals (22). Baxendale and Mebatsion (23) reported the isolation and identification of astroviruses from broiler chickens suffering from RSS and recognized them as chicken astroviruses. Currently, CAstV is distributed globally and is emerging in new geographic locations, such as Canada, China, South Korea, India, Italy, Jordan, Australia, and Brazil, in a wide range of avian species, including chickens, guinea, turkeys, fowl, and ducks (6, 22). CAstV has historically been associated with hepatitis, enteritis, nephritis, and RSS in turkeys, chickens, some mallard duck species, and wild birds (16, 24).

Notably, many researchers have proven that white chick syndrome (WCS) is caused by CAstV (6). WCS is also responsible for decreased egg production and reduction of hatchability by up to 48% of broiler breeders in the early and late periods of production (6, 21). At a young age, infected chicks were observed to have pale-to-white–colored plumage with a brown plumage ring over the neck and head, and the liver was enlarged and had a bronze or greenish color. However, in many cases, both CAstV and ANV were isolated from apparently healthy birds (25–27).

ANV was first reported and isolated from 1-week-old broiler chicks in Japan by Yamaguchi et al. (28). In 2000, it was genetically characterized and classified as an astrovirus under the family *Astroviridae* by Imada et al. (29). Currently, ANV has been reported in different poultry species in several countries, including India, the United States, Australia, Nigeria, Canada, South America, and Brazil (29–32). ANV has been associated with kidney lesions, growth suppression, and many symptoms, such as enteritis, ruffled feathers, visceral gout, and apathy, and it causes mortality in young chicks (16, 33).

Modern diagnostic methodologies have been developed to monitor flocks for CAstV and ANV using isolation and identification, detection using electron microscopy, reverse transcription polymerase chain reaction (RT-PCR), or real-time RT-PCR (rRT-PCR). Indirectly, detection of antibodies is performed using methods such as enzyme-linked immunosorbent assay (ELISA), which is costly and not available in many local markets (6). Many outbreaks of RSS with enteric disorders have also been observed over the years in Bangladesh; however, according to our review of the literature, no reports on either CAstV or ANV viruses have been published.

This study was carried out to survey the infection statutes of CAstV and ANV in different types of poultry farms located in the four districts of Bangladesh with isolation, identification, and molecular characterization of CAstV and ANV. The flocks demonstrated clinical RSS with enteric disorders, and an impact on flock mortality and FCR% in broiler chickens was demonstrated.

## Materials and Methods

### Sample Collection

**Figure 1:**
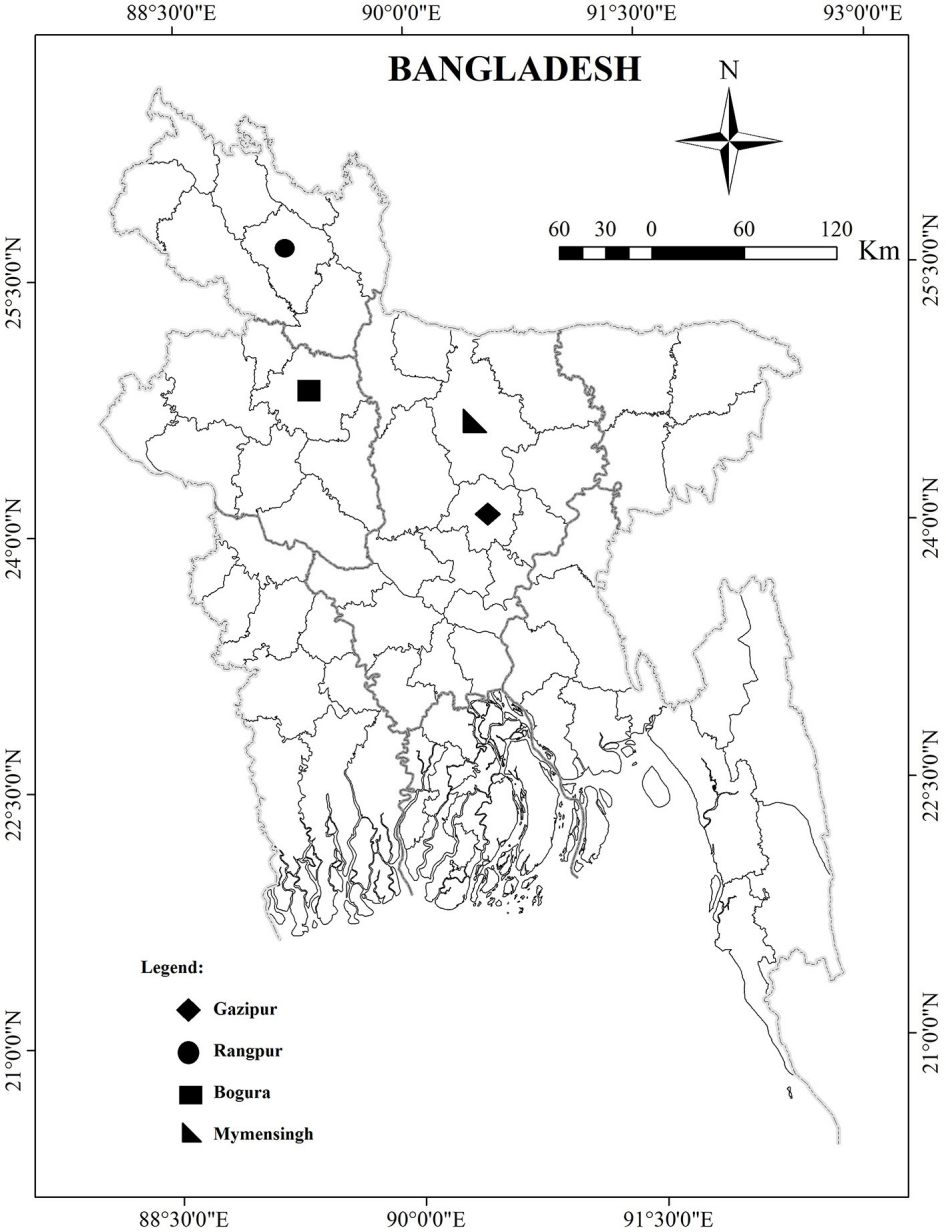
Map of Bangladesh depicting sampling location.

The investigation of CAstV and ANV was carried out in five types of chicken flocks [broilers (*n* = 18), broiler breeders (*n* = 9), layer (*n* = 2), layer breeder (*n* = 1), and indigenous (local breed) (*n* = 1)] from four districts (Gazipur, Rangpur, Mymensingh, and Bogura) of Bangladesh (Figure 1) during 2018 (Table 1). The flock had been diagnosed with enteric problems, such as diarrhea, growth retardation, poor feed conversion ratio, malabsorption syndrome, visceral gout (broiler) and articular gout (broiler breeder, layer, layer breeders and indigenous), culling, and increased mortality. Freshly dead chickens (*n* = 31) from 31 flocks were subjected to postmortem examination, and samples were collected from the kidneys, liver, duodenum, and cecum in phosphate-buffered saline (PBS) supplemented with antibiotics (penicillin: 1,000 units/ml; streptomycin: 1,000 μg/ml) and an antifungal agent (amphotericin B 2.5 μg/ml). The samples were transferred immediately to the laboratory and kept at −20°C until processing.

**Table 1:** Distribution of samples collected from five types of chickens under four areas of Bangladesh (total flock = 31).

**Sl. No**.	**Location**	**Type of chick**					**Total**
		**Broiler**	**Broiler breeder**	**Layer**	**Layer breeder**	**Indigenous**	
1	Gazipur	5	9	1	1	1	17
2	Rangpur	3	–	–	–	–	3
3	Mymensingh	3	–	1	–	–	4
4	Bogura	7	–	–	–	–	7
	Total	18	9	2	1	1	31

### Sample Preparation

The tissue samples from each flock were pooled before periodic homogenization in PBS to a final concentration of 10% (w/v), and then the supernatant was collected into 1.5-ml Eppendorf tubes by low-speed centrifugation at 4,500 × g for 15 min. Finally, the supernatant was stored at −80°C until RNA extraction. Viral RNA was extracted from 140 μl of each supernatant fluid using the QIAamp Viral RNA Mini Kit (Qiagen, Crawley, UK) according to the manufacturer's instructions.

### rRT-PCR Assays

All 31 extracted viral RNA samples were subjected to rRT-PCR assays using primers and a probe reported by Smyth et al. and described in Table 2 (17). The rRT-PCRs were set up in triplicate, and each reaction contained 20 μl of reaction mixtures prepared by 10 μl of AgPath-IDTM One-step RT-PCR 2x buffer (Applied Biosystems), 0.8 ml of AgPath-IDTM One-step RT-PCR enzyme (Applied Biosystems), a forward and reverse primer (400 nM), probe (120 nM), and 2 μl of RNA template, and nuclease-free distilled water (Applied Biosystems) was added to 20 μl. A negative control was performed by replacing the RNA template with 2 μl of nuclease-free distilled water or negative extraction control. Amplification was conducted in an ABI 7,500 fast thermal cycler (Applied Biosystems) with thermal conditions of 45°C for 10 min for the reverse transcription stage and 95°C for 10 min for the initial denaturation stage, followed by 40 cycles of denaturation at 95°C for 15 s and then annealing at 60°C for 45 sD. All samples were screened for verification of coinfection with Avian reovirus (ARV) according to the primers and protocol of Pantin-Jackwood et al. (34).

**Table 2:** Primer and probe sequences used in rRT-PCR assays for chicken astrovirus and avian nephritis virus.

**Virus**	**Primer location**	**Primer sequences**	**Product size (base pairs)**	**Reference**
Chicken astrovirus	ORF1b, ORF2	Forward: 5′-GCTGCTGCTGAAGATATACAG-3′	70	Smyth et al. (17)
		Reverse: 5′-CATCCCTCTACCAGATTTTCTGAAA-3′		
		Probe: 5′-6FAM CAGAAGTCGGGCCC BHQ1-3′		
Avian nephritis virus	3′-Untranslated region	Forward: 5′-GTAAACCACTGGTTGGCTGACT-3′	56	
		Reverse: 5′-TACTCGCCGTGGCCTCG-3′		
		Probe: 5′-6FAM CAGCAACTGACTTTC BHQ1-3′		

### Virus Isolation

Two samples were positive for CAstV but negative for ANV, and 2 ANV-positive samples, including one sample that was only ANV positive in the rRT-PCR test, were selected for isolation of CAstV and ANV. The previously processed samples for viral RNA extraction were filtered with a 0.22 μM filter and kept for inoculum. Fourteen-day-old specific pathogen-free (SPF) White Leghorn chicken embryonated eggs (CEEs) were used for virus isolation. The filtered inoculum (0.2 mL) was inoculated into the yolk sac route of SPF CEEs and sealed by melted paraffin. An inoculum of 0.2 mL of sterile PBS at pH 7.4 was inoculated through the yolk sac route of SPF CEE as a negative control. Next, inoculated CEEs were maintained in an automated egg incubator with a constant temperature of 37.5 °C to 38 °C and humidity of 40–55% for 5 days. The protocol of virus isolation was carried out according to the protocol described by Nunez et al. for CAstV and Nunez et al. for ANV (27, 35).

### Confirmation of Virus Isolation

At 5 days post infection, the growth of the virus was examined by necropsy after chilling the embryos at 4°C for 1 h. Pathological changes of the embryos, such as dwarfism, edema, gelatinous lesions, or hemorrhage, were observed. The confirmation of virus isolation was also confirmed by rRT-PCR.

### Sequencing and Molecular Analysis

Two embryos of both virus isolates confirmed by rRT-PCR were macerated, and a 1:1 suspension with PBS was made. Next, the suspensions were homogenized and centrifuged in a refrigerated centrifuge machine for 30 min at 12,000 × g. The supernatant was filtered with a 0.22 μM filter. Next, 300 μl of supernatant fluid containing cultured virus was socked in an FTA card (Merck®, Germany; maximum capacity is 500 μl) and sent for commercial Sanger sequencing. RNA was extracted from the FTA card punches, and cDNA was prepared by commercial kits and their protocols (RevertAid First Strand cDNA Synthesis Kit, Thermo Scientific). PCR was performed on both viruses with the primers and protocols described by Day et al. (36). The amplified products were purified (using ExoSAP-IT™, Applied Biosystems) and Sanger sequenced with a BigDye terminator v3.1 sequencing kit and a 3730xl automated sequencer (Applied Biosystems). The partial sequences of ORF1b were identified by a basic local alignment search tool (BLAST) search and compared with the published CAstV and ANV sequences deposited in the GenBank database. Next, the sequences were aligned and analyzed with other related sequences present in the GenBank database. The phylogenetic tree of both viruses was constructed using the software package MEGA X (37).

### Economic Parameter

Based on the tested positive broiler flocks, the mortality rate was recorded over the last 7 days starting from the day of sample collection. The feed conversion rate (FCR%) was calculated for only broilers over 30–35 days to sale or culling. The history of hatchability and egg production records of the corresponding flocks (broiler breeder, layer breeder and layer flock) to the onset of clinical examination were collected. The hatch drop and egg production drop were calculated from the difference between the onset and before the clinical examination.

### Statistics

All collected raw data were entered into a Microsoft Excel 2010 (MS Excel) spreadsheet. Next, the correlation among the variables' estimated parameters was calculated using Pearson's correlation method in SPSS (Statistical Package for the Social Sciences) software version 20 for statistical computing.

## Results

### Prevalence of CAstV and ANV

Thirty-one cases were suspected to be RSS by both standard clinical signs and postmortem lesions in five types of chickens from four districts of Bangladesh during 2018. Overall, 15/31 (48.38%) of examined cases were positive for Avastrovirus. Thus, 8/31 flocks (25.8%) were positive for CAstV, 6/31 flocks (19.3%) had mixed infection of CAstV and ANV, and 1 flock (3.2%) was positive for ANV (Table 3). CAstV was positive in all examined 4 types of chickens: 38.88% (7/18) in broilers, 44.44% (4/9) in broiler breeders, 50% (1/2) in layers, 100% (1/1) in layer breeders, and 100% (1/1) in indigenous breeders (Table 4). In the case of ANV, 33.33% (6/18) of positive cases were observed in broiler and 11.11% (1/9) broiler breeder flocks, and no positive cases were observed in the remaining three types. Out of the 15 positive cases, a mixed infection with CAstV and ANV was found in only broiler and broiler breeder flocks in 5 (5/15; 33.33%) and 1 (1/15; 6.66%) cases, respectively (Table 4). According to the location of the outbreak, comparatively, the highest number of positive cases of both single and mixed infections of CAstV and ANV were found in the Gazipur district: 8 flocks tested positive for CAstV (8/17; 47.05%), 3 flocks tested positive for ANV (3/17; 17.65%) and 2 flocks tested positive (2/17; 11.76%) for mixed infection. For ANV, positive cases were found in 2 (2/3; 66.66%) of the Rangpur districts and 2 (2/4; 50%) of the Mymensingh districts, but no cases were identified in the Bogura district. In addition, mixed infection cases were not found in poultry flocks in the Bogura district (Table 5). No Avian reovirus coinfection was identified in the tested samples.

**Table 3:** Identification of CAstV and ANV in chickens in selected areas of Bangladesh (total flock = 31).

**Flock**	**Location**	**Age**	**Type of chicken**	**Flock Mortality^**a**^ (%)**	**FCR %**	**Hatch drop %**	**Egg production drop %**	**rRT-PCR results**		
								**CAstV**	**ANV**	**Comments**
F10	Gazipur	29 W	Broiler breeder	0	–	5.2^*^	12.23^*^	Neg	Neg	–
F11	Gazipur	27 W	Broiler breeder	0	–	7.06^*^	9.83^*^	Neg	Neg	–
F12	Gazipur	45 W	Broiler breeder	0	–	5.42^*^	16.51^*^	Neg	Neg	–
F13	Gazipur	41 W	Broiler breeder	0	–	3.22^*^	8.64^*^	Neg	Neg	–
F14	Gazipur	30 W	Broiler breeder	0	–	7.21^*^	8.41^*^	Neg	Neg	–
F15	Gazipur	30 W	Broiler breeder	2.85	–	23.67^**^	39.4^**^	Pos	Pos	CAstV, ANV
F17	Gazipur	25 W	Broiler breeder	3.11	–	18.16^**^	22.62^**^	Pos	Neg	CAstV
F18	Gazipur	46 W	Layer breeder	2.04	–	15.33^**^	24.81^**^	Pos	Neg	CAstV
F19	Gazipur	42 W	Broiler breeder	3.85	–	17.06^**^	20.39^**^	Pos	Neg	CAstV
F20	Gazipur	32 W	Broiler breeder	2.77	–	20.52^**^	26.3^**^	Pos	Neg	CAstV
F1	Mymensingh	24 D	Broiler	7.29	1.83	–	–	Pos	Pos	CAstV, ANV
F2	Gazipur	25 D	Broiler	6.89	2.23	–	–	Neg	Pos	ANV
F3	Bogura	25 D	Broiler	3.44	1.55	–	–	Neg	Neg	–
F4	Mymensingh	21 D	Broiler	5.61	1.9	–	–	Pos	Pos	CAstV, ANV
F5	Bogura	25 D	Broiler	7.89	2.06	–	–	Pos	Neg	CAstV
F6	Gazipur	28 D	Broiler	4.01	1.62	–	–	Neg	Neg	–
F7	Mymensingh	23 D	Broiler	3.23	1.68	–	–	Neg	Neg	–
F8	Gazipur	21 D	Broiler	4.07	1.8	–	–	Neg	Neg	–
F9	Gazipur	29 D	Broiler	2.33	1.48	–	–	Neg	Neg	–
F16	Rangpur	16 D	Broiler	1.5	1.92	–	–	Pos	Neg	CAstV
F21	Gazipur	13 D	Broiler	5.08	2.17	–	–	Pos	Pos	CAstV, ANV
F24	Rangpur	24 D	Broiler	4.17	1.9	–	–	Pos	Pos	CAstV, ANV
F25	Rangpur	24 D	Broiler	4.06	1.85	–	–	Pos	Pos	CAstV, ANV
F27	Bogura	23 D	Broiler	4.44	1.77	–	–	Neg	Neg	–
F28	Bogura	25 D	Broiler	3.93	1.79	–	–	Neg	Neg	–
F29	Bogura	29 D	Broiler	4.08	1.6	–	–	Neg	Neg	–
F30	Bogura	28 D	Broiler	2.87	1.82	–	–	Neg	Neg	–
F31	Bogura	21 D	Broiler	4.6	1.85	–	–	Neg	Neg	–
F22	Gazipur	22 W	Layer	2.46	–	10.21^**^	27.31^**^	Pos	Neg	CAstV
F26	Mymensingh	11 W	Layer	3.74	–	–	–	Neg	Neg	–
F23	Gazipur	13 D	Indigenous	1.72	–	–	–	Pos	Neg	CAstV

* *Significant at the 0.05 level*;

** *Significant at the 0.01 level*.

*CAstV, Chicken astrovirus; ANV, Avian nephritis virus*.

a *The mortalities were recorded for 7 days from sampling day*.

**Table 4:** Identification of CAstV and ANV in types of chickens.

**Type of Chicken**	**rRT-PCR results**		
	**CAstV**	**ANV**	**CAstV+ANV**
Broiler (*n* = 18)	2 (11.11%)	–	5 (27.77%)
Broiler breeder (*n* = 9)	3 (33.33%)	1 (11.11%)	1 (11.11%)
Layer (*n* = 2)	1 (50%)	–	–
Layer breeder (*n* = 1)	1 (100%)	–	–
Indigenous (*n* = 1)	1 (100%)	–	–
P	0.015^*^	0.000^**^	0.11^*^
Total	8 (25.81%)	1 (3.22%)	6 (19.35%)

* *Significant at the 0.05 level*;

** *Significant at the 0.01 level*.

*CAstV, Chicken astrovirus; ANV, Avian nephritis virus*.

**Table 5:** Identification of CAstV and ANV on location of chicken flocks.

**Location**	**rRT-PCR results**		
	**CAstV**	**ANV**	**CAstV+ANV**
Gazipur (*n* = 17)	6 (35.29%)	1 (5.88%)	2 (11.76%)
Rangpur (*n* = 3)	1 (33.33%)	–	2 (66.66%)
Mymensingh (*n* = 4)	–	–	2 (50.00%)
Bogura (*n* = 7)	1 (14.28%)	–	–
P	0.013^**^	0.001^**^	0.034^*^
Total	8 (25.81%)	1 (3.22%)	6 (19.35%)

* *Significant at the 0.05 level*;

** *Significant at the 0.01 level*.

*CAstV, Chicken astrovirus; ANV, Avian nephritis virus*.

### Isolation of CAstV and ANV

**Figure 2:**
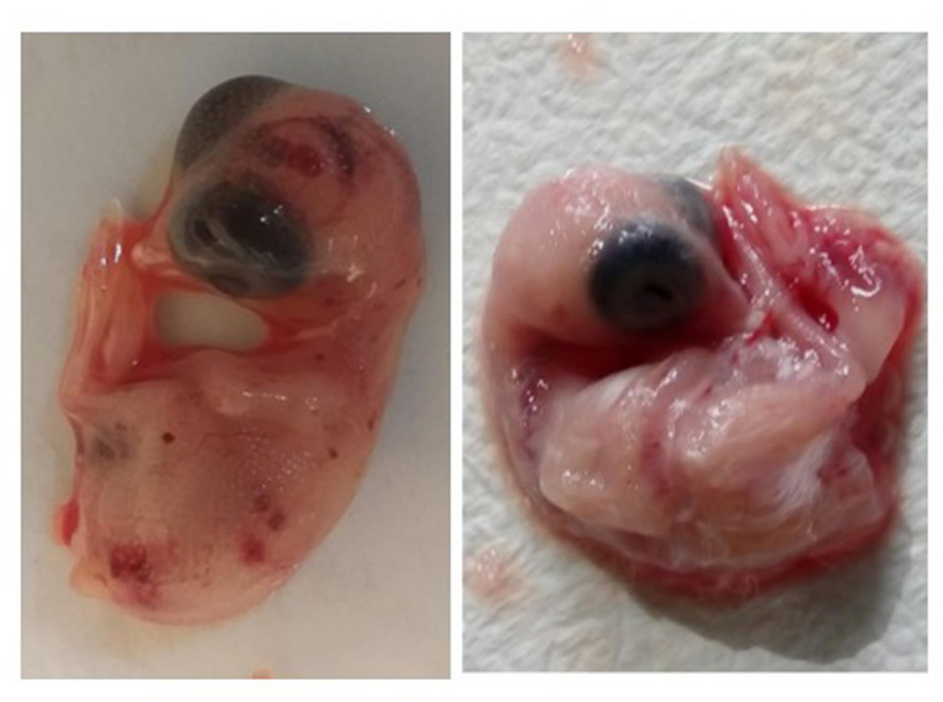
The hemorrhagic, edematous chicken embryos with gelatinous lesions and dwarfism due to growth of virus after inoculation of virus through yolk sac route (left image) and non-infected chicken embryo (right image).

In this study, only two isolates from both viruses were cultured in SPF CEE because of the unavailability of SPF chicken eggs. Here, both the CAstV and ANV of each of the two isolates were shown to have growth in lesions observed after one passage that continued to the second passage (Figure 2). Next, the virus isolates were confirmed using the rRT-PCR test. No pathological lesions on embryos were observed in the negative controls for either virus.

### Sequencing of CAstV and ANV

**Figure 3:**
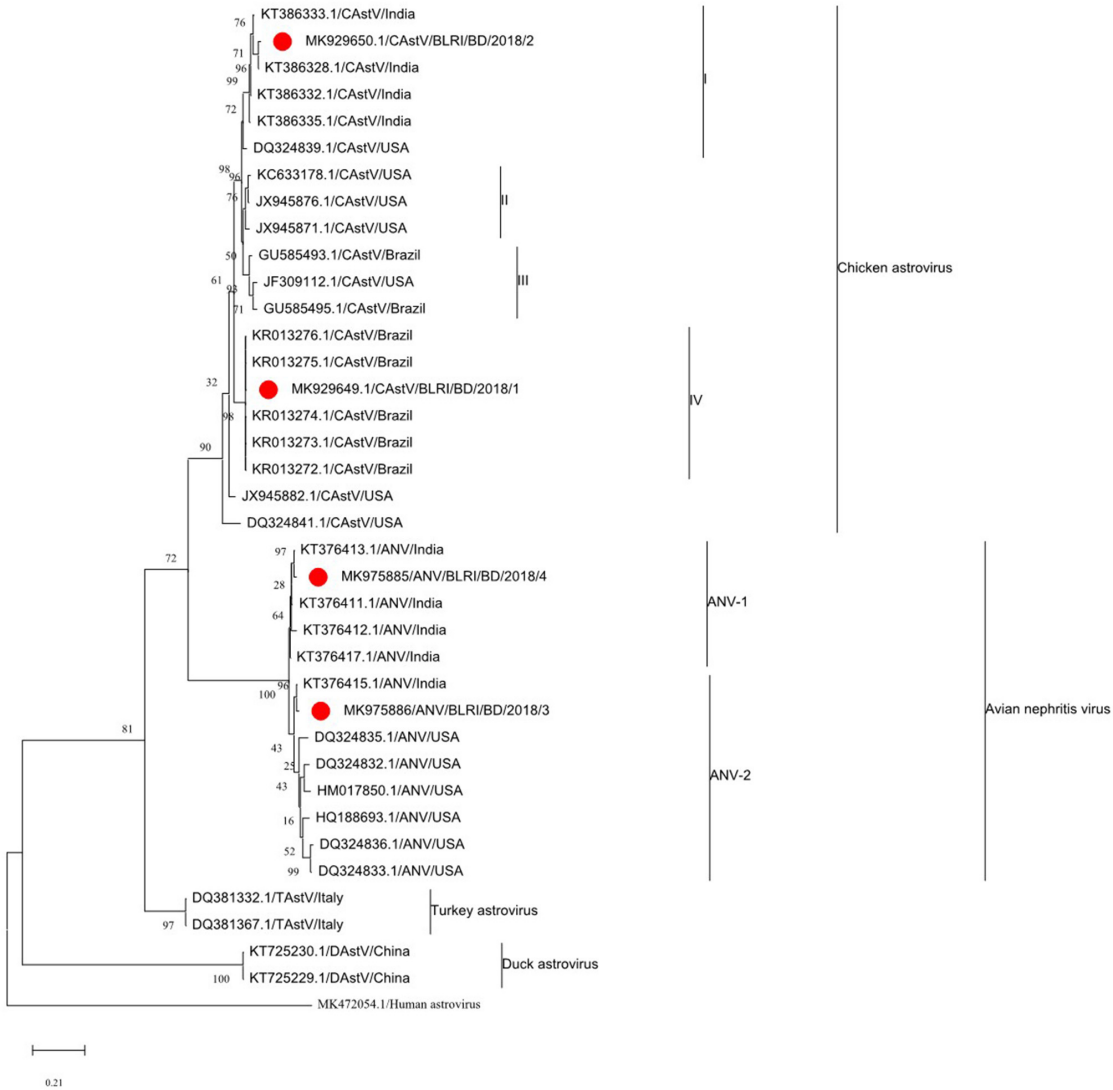
The phylogenetic tree was constructed using MEGA X software on the alignments of the partial ORF1b sequences of CAstV (362 nucleotide length)and ANV (473 nucleotide length) using a neighbor-joining phylogeny method joined with the maximum composite likelihood model with 1,000 bootstraps of replication. The tree showed the phylogenetic relationships of the Bangladeshi CAstV and ANV isolates in SPF CEEs with other sequences present in GenBank. The numbers along the branches show the bootstrap value for every 1,000 replicates. The scale bar represents the number of substitutions per site. The GenBank accession numbers and host species of the sequences used here are shown in the tree. The red color with bold sequences is Bangladeshi isolates. The human astrovirus 1 sequence was used as an outgroup.

The obtained partial nucleotide sequence of the ORF1b gene of four isolates (two of each virus) was submitted to the NCBI GenBank under accession numbers MK929649.1 (CAstV/BLRI/BD/2018/1), MK929650.1 (CAstV/BLRI/BD/2018/2), MK975886.1 (ANV/BLRI/BD/2018/3), and MK975885.1 (ANV/BLRI/BD/2018/4). The Basic Local Alignment Search Tool (BLAST) search revealed that 2 CAstV sequences of Bangladesh origin were similar to the CAstV sequences of Brazil (MK929649.1 is 99.45% with Brazilian KR013276.1) and India (MK929650.1 is 99.18% with Indian KT386328.1). Another two sequences of ANV of Bangladesh origin were closely related to an ANV-1 strain and similar to an ANV sequence from India (MK975886.1 and MK975885.1 are 98.90% similar to KT376415.1 and KT376413.1, respectively). Notably, the in-between sequences of CAstV and ANV are identical. The relationships among the sequences were described by a phylogenetic tree (Figure 3).

### Economic Impact of CAstV and ANV

Broiler flocks recorded significantly lower FCR%, and a higher mortality rate was observed in the flocks infected with both CAstV and ANV compared with infection with a single virus. The lowest flock mortality (1.50%) was in flock number F16, which had a better FCR% (1.83%) than being infected by only CAstV (Table 3). A significant correlation was observed among flock mortality (%), FCR%, and infection with CAstV and ANV for the selected flocks. The hatch drop and egg production drop were found to be significantly higher in flocks infected with CAstV and ANV.

## Discussion

The cause of RSS in poultry is multifactorial (3, 32, 38). RSS symptoms are observed regularly by poultry veterinarians in Bangladesh, but no investigations have been carried out to determine the role of CAstV or ANV as viral agents in this syndrome.

Many virologic confirmations have been established to diagnose CAstV and ANV, such as isolation by cell culture or embryo inoculation, but this process is complicated and time-consuming for screening an entire flock, and using specific antisera for identification of a virus is also complicated (39, 40). However, rRT-PCR and conventional RT-PCR are highly sensitive and specific as well as rapid and compatible for screening and diagnosis of clinical samples (22, 26, 27, 39–41).

In this investigation, real-time RT-PCR was used to detect viral RNA of both CAstV and ANV in pooled samples of liver, kidney, and intestine (duodenum, cecum) from clinically suspected poultry flocks. Many researchers have identified both CAstV and ANV in a wide range of samples, such as liver, kidney, cecum, duodenum, intestinal contents, and yolk, and found a comparatively higher concentration of ANV RNA in intestinal or gut samples (10, 11, 27, 32, 39–42).

This study confirmed that 48.38% (15/31) of flocks were positive for CAstV and ANV either as a single infection or mixed infection in broilers, broiler breeders, layers, layer breeders, and indigenous chickens in different districts in Bangladesh. The results indicate that broiler flock mortality rates were increased in flocks with a mixed infection compared with flocks with a single infection. Similar observations in South Korea were published by Koo et al., who surveyed South Korean poultry farms with enteric disorders and reported that seven enteric viruses with three bacterial coinfections were involved (26). They demonstrated that the flocks had higher rates of infection with ANV (44.1%) than with CAstV (38.2%), and 51.7% of cases were infected with at least two or more enteric viruses. In contrast, Oluwayelu et al. reported a higher prevalence compared with our experience, namely, 92.3% of ANV and 53.9% of CAstV in Nigerian indigenous chickens (42). Another survey was conducted in the United States that isolated CAstV from 90% healthy as subclinical infection and 100% from an RSS-reported chicken flock when intestinal contents were tested by rRT-PCR (4).

Mixed infection with multiple enteric viruses and bacteria has been demonstrated to have a more severe effect and prolonged enteritis than infection with a single virus in poultry (4, 26). Therefore, multiple infections increase flock mortality rates and decrease the FCR to a greater extent in broiler-type chickens (6). They are also risk factors for secondary infections with opportunistic pathogens such as *Escherichia coli, Salmonella* spp., and *Eimeria* spp., which disrupt the normal physiology of the enteric mucosa. Thus, impaired nutrition absorption and impaired development of primary defense organs such as the thymus and bursa of Fabricius results in chicks suffering from different malabsorption syndromes, such as RSS (2, 26). CAstV infection in broiler breeder flocks significantly decreased egg production up to 21% and decreased hatchability rates up to 68.4% (43). Broilers are the most susceptible to CAstV and ANV, resulting in an increased mortality of up to 40% in their first week of age and interstitial nephritis and gout (12). Chickens in the first 3 weeks of life are more susceptible to simultaneous infection with enteric viruses CAstV, ANV, and ARV (22, 26). We could not confirm articular gout is mainly by CAstVs or not but that was our findings on most of chickens that confirmed CAstVs cases and we suggested further studies on correlation between CAstVs and articular gout.

In this study, four types of commercial poultry production and one indigenous chicken flock were infected with CAstV where most of the broiler chickens infected with combined of CAstV and ANV. This indicates that these viruses are widely circulating and are probably endemic throughout the country, which should be alarming to the poultry industry. The actual prevalence of this virus in Bangladeshi poultry industries may be higher than that in this study, since the extent of spread of avian enteric viruses remains unclear.

CAstV and ANV can grow well in the CEE through the yolk sac route. Inoculation of a virus through the yolk sac route in 7-day-old embryonated eggs is the standard method for virus isolation (22). In contrast, in this study, 14-day-old embryonated eggs were inoculated because of the enteric nature of CAstV and ANV and because the intestine would already be developed and functional at this stage of embryo development, which may improve virus isolation (44).

Genetically, the sequence of the ORF1b conserved region of CAstV and ANV confirmed that the viruses were the causal agents of RSS and circulated in chickens. Analysis of the nucleic acid sequences of this study revealed their similarity with reported viruses from India and other parts of the world (45). All CAstV and ANV of this study were clustered in a group of ORF1b polymerase gene phylogeny. Kaithal et al. (46) analyzed the ORF1b genes of CAstV and ANV and revealed that Indian isolates of CAstV and ANV were aligned with circulating and Brazilian sequences of the respective viruses. It could be assumed that transboundary transmission of both viruses through neighboring borders may occur. Further ORF2 sequence analysis is recommended to investigate the antigenic variation of our isolates. Cloning and whole genome sequencing of CAstV and ANV is essential to extend genetic diversity and to discover genetic variations in diverse poultry species (45). Consequently, differential diagnosis in a poultry flock with decreased FCR, enteritis, poor hatchability, and retarded growth should consider RSS caused by CAstV and ANV.

CAstV and ANV infection directly impact egg production drops and hatchability drops in broiler breeder flocks. Hatchability losses are prominent during mid-period and late-period incubation, and they can be caused by the chicks being too weak to hatch out or slow to hatch (17, 47, 48). In this study, a significant correlation was found between egg production drop and hatchability drop with flocks positive for CAstV and ANV.

## Conclusion

This is the first report of molecular evidence of chicken astrovirus (CAstV) and avian nephritis virus (ANV) in chickens with runting-stunting syndrome in Bangladesh. The sequencing results confirmed that the identified CAstV in Bangladesh is similar to strains isolated from Brazil and India. The ANV is grouped with ANV-1 and is closely related to strains isolated from India. Our results will be useful for diagnosis and vaccine development for both CAstV and ANV in Bangladesh. More extensive epidemiological studies, whole genome sequencing and pathogenicity studies of circulating strains are recommended.

## Data Availability Statement

The raw data supporting the conclusions of this article will be made available by the authors, without undue reservation.

## Ethics Statement

The animal study was reviewed and approved by The Animal Experimentation Ethics Committee approval number: BLRI0005.

## Author Contributions

HJS and MZA supervised this project, designed the experiments, and prepared manuscript. MMM, ZAB, and MG collected samples and performed experiments. All authors read and approved the final manuscript.

## Funding

This work was supported by the Chungnam National University, South Korea.

## Conflict of Interest

MMM and ZAB are employed by Nourish Central Poultry Laboratory, Nourish Poultry and Hatchery Ltd., Dhaka, Bangladesh. The remaining authors declare that the research was conducted in the absence of any commercial or financial relationships that could be construed as a potential conflict of interest.

## Publisher's Note

All claims expressed in this article are solely those of the authors and do not necessarily represent those of their affiliated organizations, or those of the publisher, the editors and the reviewers. Any product that may be evaluated in this article, or claim that may be made by its manufacturer, is not guaranteed or endorsed by the publisher.
